# Physical Activity, Mental Health, and Technology Preferences to Support Cancer Survivors During the COVID-19 Pandemic: Cross-sectional Study

**DOI:** 10.2196/25317

**Published:** 2021-02-03

**Authors:** Jamie M Faro, Kristin M Mattocks, Catherine S Nagawa, Stephenie C Lemon, Bo Wang, Sarah L Cutrona, Rajani S Sadasivam

**Affiliations:** 1 Division of Health Informatics and Implementation Science Department of Population and Quantitative Health Sciences University of Massachusetts Medical School Worcester, MA United States; 2 VA Central Western Massachusetts Healthcare System Northampton, MA United States; 3 Division of Preventive Behavioral Medicine Department of Population and Quantitative Health Sciences University of Massachusetts Medical School Worcester, MA United States; 4 Biostatistics and Health Services Research Department of Population and Quantitative Health Sciences University of Massachusetts Medical School Worcester, MA United States; 5 Center for Healthcare Organization and Implementation Research Edith Nourse Rogers Memorial Veterans Hospital Bedford, MA United States

**Keywords:** cancer, COVID-19, digital, physical activity, support, technology

## Abstract

**Background:**

COVID-19 has had significant health-related and behavioral impacts worldwide. Cancer survivors (hereafter referred to as “survivors”) are particularly prone to behavioral changes and are encouraged to be more vigilant and observe stricter social distancing measures.

**Objective:**

We explored (1) changes in physical activity and sedentary behaviors since the onset of COVID-19, along with changes in mental health status, and (2) alternative strategies to support survivors’ physical activity and social health during and after COVID-19, along with the role of digital health in such strategies.

**Methods:**

A questionnaire was distributed among survivors participating (currently or previously) in the community-based physical activity program LIVESTRONG at the Young Men’s Christian Association (YMCA), from 3 sites outside an urban area in Massachusetts. Questions addressed pre–COVID-19 vs current changes in physical activity and sedentary behavior. Anxiety and depression were assessed using the 2-item Generalized Anxiety Disorder scale (GAD-2) and 2-item Patient Health Questionnaire (PHQ-2), and scores ≥3 indicated a clinical diagnosis of anxiety or depression, respectively. Digital health preferences were assessed through closed-ended questions. Open-ended responses addressing other preferences for physical activity programs and social support were analyzed, coded, and categorized into themes.

**Results:**

Among 61 participants (mean age 62 [SD 10.4] years; females: 51/61 [83.6%]), 67.2% (n=41) reported decreased physical activity and 67.2% (n=41) reported prolonged sitting times since the onset of COVID-19. Further, 24.6% (n=15) and 26.2% (n=16) met the GAD-2 and PHQ-2 criteria for clinical anxiety and depression, respectively. All participants owned a cellphone; 90% (n=54) owned a smartphone. Preferences for physical activity programs (n=28) included three themes: (1) use of digital or remote platforms (Zoom, other online platforms, and video platforms), (2) specific activities and locations (eg, outdoor activities, walking, gardening, biking, and physical activities at the YMCA and at senior centers), and (3) importance of social support regardless of activity type (eg, time spent with family, friends, peers, or coaches). The survey revealed a mean score of 71.8 (SD 21.4; scale 0-100) for the importance of social support during physical activity programs. Social support preferences (n=15) revealed three themes: (1) support through remote platforms (eg, texting, Zoom, phone calls, emails, and Facebook), (2) tangible in-person support (YMCA and senior centers), and (3) social support with no specific platform (eg, small gatherings and family or friend visits).

**Conclusions:**

Physical activity and mental health are critical factors for the quality of life of survivors, and interventions tailored to their activity preferences are necessary. Digital or remote physical activity programs with added social support may help address the ongoing needs of survivors during and after the pandemic.

## Introduction

COVID-19 first emerged in December 2019 [1]. COVID-19 and social distancing have had deleterious effects on physical activity and mental health in the general population, resulting in decreased activity levels and increased anxiety, depression, and stress levels [2,3]. Current cancer survivors (hereafter referred to as “survivors”) and those previously undergoing treatment may have been affected in particular. Survivors have unique emotional needs owing to anxiety, depression, and familial and financial strains, along with many long-lasting preexisting health conditions [4-7]. However, few studies have addressed these concerns and explored means to provide additional support to survivors. Owing to their preexisting conditions and immunocompromised state, survivors are at an increased risk of disease and admission to the intensive care unit, increased ventilator use, and an increased risk of death due to COVID-19 [8,9]. Hence, survivors are encouraged to observe strict social distancing guidelines [10]. Further, many in-person survivorship resources, such as physical activity and mental health support, have been reduced [11].

We explored the effects of COVID-19 on a group of survivors who were current or previous participants in the community-based physical activity program, LIVESTRONG at the Young Men’s Christian Association (YMCA) [12]. This 12-week program involves physical activity (ie, aerobic, muscle strengthening, and flexibility exercises) and social support (ie, group support sessions) delivered in person by trained staff, twice a week, free of cost to survivors at participating YMCA sites nationwide. The program has effectively improved survivors’ physical activity, fitness, and quality of life [13]. In this study, we examined (1) changes in physical activity or sedentary behaviors since the onset of COVID-19, along with changes in their mental health status, and (2) alternative strategies to support survivors’ physical activity and social health during and after COVID-19, including the role of digital health in these strategies. Although the role of digital health in promoting physical activity and mental health has been understudied among survivors [14], some trials [15] have reported the feasibility, adherence, and effectiveness of digital health [15]. Because the lasting effects of COVID-19 are unknown, this formative study may contribute to the development of digital community-based physical activity and social support programs.

## Methods

### Study Design

This cross-sectional study included individuals participating in the 12-week LIVESTRONG at the YMCA program, which delivers support for physical activities and social health free of cost for those who (1) have or have had a cancer diagnosis, (2) are over 18 years of age, and (3) were medically cleared by a physician to perform physical activity. We coordinated with the program director at one LIVESTRONG site, who contacted program directors at two additional local sites outside an urban area in Massachusetts, to describe the study and deliver an online questionnaire survey to current and past program participants. From among these three sites, we estimated that these listservs had >300 eligible participants, but we could not estimate the total number of emails sent. Participants were provided a US $10 gift card upon completion of the questionnaire. The Institutional Review Board at UMass Medical School approved this trial (IRB docket number H00020448).

### Measures

#### Physical Activity and Sedentary Time

We assessed subjective changes in physical activity by asking the question, “Since COVID-19, has your physical activity (a) decreased, (b) increased, or (c) stayed the same?” We assessed changes in sedentary behaviors by asking the question, “Since COVID-19, has your time spent sitting (a) decreased, (b) increased, or (c) stayed the same?”

#### Mental Health

Anxiety and depression were assessed using the 2-item Generalized Anxiety Disorder scale (GAD-2) [16] and the 2-item Patient Health Questionnaire (PHQ-2) [17], respectively. Both these tools have acceptable sensitivity and specificity [18].

#### Physical Activity During or After COVID-19 and Digital Health Preferences

Participants were asked to report all their preferred physical activities during and after COVID-19 from among the following: (1) indoor or outdoor activities with family or friends, (2) indoor or outdoor activities by themselves, (3) physical activity delivered through online platforms, and (4) physical activity delivered through video calls with family, friends, or fitness professionals. They were then asked to respond to an optional open-ended question regarding other preferred means of receiving physical activity programs.

#### Social Support and Digital Health Preferences

Participants ranked the importance of social support in a physical activity program (scale 0-100). They reported their most preferred means of receiving social support from among the following: (1) in person, (2) video calls, (3) social media, and (4) texting. They then responded to open-ended questions regarding their preferred means of receiving social support.

### Statistical Analysis

We analyzed descriptive statistics for quantitative variables, using STATA (version 15, StataCorp). For the GAD-2 and PHQ-2, we summed the two questions and applied a cut-off ≥3 to generate a dichotomous variable. Scores ≥3 were classified as “clinically diagnosable” independently for anxiety and depression [16,17]. Responses to open-ended questions were open-coded verbatim to identify relevant themes and corroborated with two additional investigators.

## Results

Participants (N=61) had a mean age of 62 (SD 10.4) years, were mainly female (n=51, 83.6%), and had pursued higher education (college diploma: n=18, 29.5%; bachelor’s degree: n=15, 24.6%; advanced college degree: n=24, 39.3%). All of them owned a cell phone, and the vast majority (n=54, 90%) owned smartphones that can access the internet (Table 1).

**Table 1:** Demographic characteristics and technology usage among the study participants (N=61).

Variable		Value
**Gender, n (%)**		
	Male	10 (16.4)
	Female	51 (83.6)
Age (years), mean (SD)		62.0 (10.4)
**Marital status, n (%)**		
	Married	38 (62.3)
	Divorced/separated	10 (16.4)
	Widowed	5 (8.2)
	Single/unmarried	8 (13.1)
**Education level, n (%)**		
	Finished high school or GED^a^	4 (6.6)
	College diploma	18 (29.5)
	Bachelor’s degree	15 (24.6)
	Advanced college degree	24 (39.3)
Uses the internet, n (%)		55 (90.2)
**How do you use the internet?, n (%)**		
	Read information on websites	55 (90.2)
	Send or receive emails	35 (57.4)
	Watch videos/listen to audio clips	21 (34.4)
	Use online social network sites	40 (65.6)
Owns a cell phone, n (%)		61 (100)
Owns a smartphone, n (%)		54 (90)
**How do you use your cell phone?, n (%)**		
	Send or receive emails	39 (63.9)
	Send or receive text messages	45 (73.8)
	Access the internet	42 (68.9)
	Look for health/medical information online	36 (61)
	Take photographs	46 (78)

^a^GED: General Education Diploma.

### Physical Activity, Sedentary Time, and Mental Health

Most participants reported decreased physical activity (n=41, 67.2%) and a prolonged sitting time (n=41, 67.2%) since the onset of COVID-19 (Table 2). On mental health evaluation, 26.2% (n=16) and 24.6% (n=15) of participants had scores greater than the clinical cut-off for depression and anxiety, respectively.

**Table 2:** Changes in physical activity and sedentary time, mental health evaluation, and preferences for physical activity and social support among the study participants (N=61).

Variable		Value
**Change in physical activity^a^, n (%)**		
	More physically active	13 (21.3)
	No change in physical activity	7 (11.5)
	Less physically active	41 (67.2)
**Change in sedentary time^a^, n (%)**		
	Sitting more	41 (67.2)
	No change in sitting time	16 (26.2)
	Sitting less	4 (6.6)
**PHQ-2^b^ score, mean (SD)**		1.35 (1.4)
	<3, n (%)	45 (73.8)
	≥3 (clinical cut-off), n (%)	16 (26.2)
**GAD-2^c, mean (SD)^**		1.84 (1.53)
	<3, n (%)	46 (75.4)
	≥3 (clinical cut-off), n (%)	15 (24.6)
**Physical activity preference during COVID-19^d^, n (%)**		
	Online programs	17 (27.9)
	Indoor or outdoor activities with family or friends	26 (42.6)
	Indoor or outdoor activities by themselves	40 (65.6)
	Video calls (with family, friends, or fitness professionals)	25 (42.6)
**Physical activity preference after COVID-19^d^, n (%)**		
	Online programs	31 (50.8)
	Indoor or outdoor activities with family or friends	24 (39.3)
	Indoor or outdoor activities by self	24 (39.3)
	Video calls (with family, friends, or fitness professionals)	14 (23)
Importance of social support for physical activity programs (scale 0-100), mean (SD)		71.8 (21.4)
**Social support preference, n (%)**		
	In-person	42 (68.9)
	Video calls	13 (21.3)
	Social media groups	3 (4.9)
	Texting	3 (4.9)

^a^Questions addressing variables for comparison with pre–COVID-19 values.

^b^PHQ-2: 2-item Patient Health Questionnaire.

^c^GAD-2: 2-item Generalized Anxiety Disorder.

^d^Participants were asked to check all applicable responses.

### Physical Activity During or After COVID-19 and Digital Health Preferences

Table 2 highlights the preferred physical activities during and after COVID-19. During COVID-19, survivors most preferred indoor activities by themselves (n=40, 65.6%) in person; after COVID-19, online programs (n=31, 50.8%). Three main themes were identified from the open-ended responses (n=28) regarding the survivors’ preferences for other physical activity programs. The present themes and sample responses are provided below.

#### Digital and Remote Programs

One participant who was in enrolled in the LIVESTRONG program stated the following when the program was moved to an online platform: “I like the Zoom program better than anything I have ever done.” Three others (10.7%) reported the following regarding remote activities and the use of technology: “Challenges on Fitbit,” “online,” and “videos or DVDs.”

#### Specific Activities or Specific Locations for Activities

In total, 4 (14.3%) survivors preferred “walking, light hiking,” “walking trails,” and “swimming” at no specified location, 6 (25%) others preferred additional outdoor activities including “outdoor activities - walking, biking,” “bike riding, fishing, gardening,” “yard work,” and “anything outdoors,” and 4 (16.7%) participants preferred indoors “gym,” “senior center,” “LIVESTRONG,” and “market walking.”

#### Importance of Social Support Regardless of Activity Type

In total, 6 (25%) participants preferred social support in addition to their preferred physical activity, such as “phone call with a friend while walking ‘together’” and “walking with friends, family, or other people,” “gym with cancer patients,” and “I need a partner to hold me accountable.”

### Preferences for Alternative Means of Social Support

Table 2 highlights the participant preferences for social support and digital health. Three main themes were identified from the questions on open-ended preferences (n=15) for other forms of social support.

#### Using Digital Platforms for Support

In total, 5 (33.3%) participants preferred social support to be delivered through a remote or digital platform, such as “phone calls, emails, Zoom, texting, Google, Nest, WhatsApp, and Facebook.” Others (n=2, 13.3%) indicated the involvement of specific individuals, such as “text messages with peers or coaches,” and “video conferencing or phone calls with friends or family.”

#### Tangible In-Person Support

In total, 3 (20%) participants preferred in-person support (“I would like to have support in person, but [the Y] is just too far away from my house…” and “to attend senior center,” “Gym or fitness center”).

#### Social Support With no Specified Platform

In total, 5 (33.3%) participants preferred social support but did not specify whether they preferred in-person or remote support (“an advocate to help with the things I struggle with,” “visits with friends or family,” and “small groups”).

### Discussion

#### Principal Findings

Survivors self-reported decreased physical activity levels and greater anxiety and depression levels, similar to those of the general population [2]. In both our quantitative and qualitive analyses, survivors reported their preferences for digital health. Other reported preferences highlighted the importance of social support.

Reductions in the survivors’ physical activity and increases in sedentary behaviors are concerning, as physical activity is critical for their physical and mental health [19-21]. The proportions of our participants who met the diagnostic criteria for depression and anxiety (n=16 [26.2%] and n=15 [24.6%], respectively) were higher than those previously reported for prostate cancer survivors (9.4% and 7.9%, respectively) but lower than those of breast cancer survivors (32.2% and 38.2%, respectively) [22,23]. Overall proportions of survivors are much higher than those of the general US population (3.1% of the general US population adults >18 years have been diagnosed with generalized anxiety disorder and 7.1% with major depressive disorder) [24]. It is critical to develop methods to support the mental health needs of survivors, and our study suggests that the pandemic and social isolation may increase the need for such support.

Survivors expressed their interest in support from digital health platforms for physical activities after COVID-19. While more survivors (n=40, 65.6%) reported their preferences for indoor or outdoor activities by themselves during COVID-19, they may possibly not have had the opportunity to receive support from digital health programs thus far. During and after COVID-19, digital health platforms may serve as a substitute for in-person support for survivors, and they can be tailored to augment the benefits of in-person support, potentially providing long-term support to survivors. COVID-19 has forced practitioners and survivors to embrace digital technology to promote and maintain health care provision and deliver physical activity programs [25]. Some oncological trials assessing physical activity have shifted to digital platforms [26]; however, community-based programs may provide less support and resources to convert in-person programs to virtual ones. A worldwide survey of fitness trends in 2021 reported that online programs ranked 1st in 2021 as opposed to 26th in 2020 [27], indicating that COVID-19 has brought about a paradigm shift in the fitness industry, and practitioners will need to adjust to this trend.

Furthermore, survivors acknowledged the importance of social support, and some expressed preferences for peer support. Telephonically delivered support by trained peer coaches coupled with remote activity monitoring has led to an increase in physical activity in a randomized trial with breast cancer survivors [28]. Although, in this study, 69% (n=42) of survivors preferred in-person social support, peer support for survivors delivered digitally is ideal for those who are unable to attend in-person programs and need additional support [29]. A digital health intervention that includes social support (ie, from family or friends, peer coaches, or other survivors) may address the preferences of survivors for remote programs and their accountability, while potentially improving their physical activity and mental health [30]. However, larger trials are required to examine the causal effects of different types of social support among survivors [31].

#### Limitations

This exploratory study had a small cohort size; therefore, the statistical power was not high enough to enable hypothesis testing to assess relationships among variables. Survivors having already participated in the LIVESTRONG at the YMCA program might have been more motivated than those not enrolled in this program; thus, reductions in physical activity reported here may underestimate the prevalence of this issue in the general survivor population. Although this study lacks data on cancer types, previous studies have reported data from a higher proportion of breast cancer survivors [13]. These studies and our study show the homogeneity of the characteristics of participants in the LIVESTRONG to the YMCA program [13], and future trials will need to examine more diverse populations. Program directors had access to email listservs through the YMCA but did not have access to the number of participants registered on them; hence, we could not determine neither the final number of emails sent to eligible participants nor the valid response rate. All responses are self-reported and were obtained in a cross-sectional manner, warranting future assessments of baseline measures to assess longitudinal changes objectively, for example, using activity monitors.

#### Conclusions

In conclusion, during and after COVID-19, survivors may benefit from support to sustain their physical activity levels and mental health. The survivors in our study voiced various preferences for physical activity and social support, some preferring indoor physical activity by themselves during COVID-19 and others preferring online or remote programs along with social support, including support from family or friends and peer coaches, after COVID-19. Community-based physical activity programs can successfully engage survivors if they provide programs tailored to individual preferences during and after the COVID-19 pandemic.

## Abbreviations

GAD-2: 2-item Generalized Anxiety Disorder
PHQ-2: Patient Health Questionnaire
YMCA: Young Men’s Christian Association
